# Family planning service disruptions in the first two years of the COVID-19 pandemic: Evidence from health facilities in seven low- and middle-income countries

**DOI:** 10.1371/journal.pgph.0002435

**Published:** 2024-01-05

**Authors:** Celia Karp, Kelsey Williams, Shannon N. Wood, Funmilola M. OlaOlorun, Pierre Akilimali, Georges Guiella, Peter Gichangi, Rosine Mosso, Frederick Makumbi, Philip A. Anglewicz, Caroline Moreau

**Affiliations:** 1 Department of Population, Family and Reproductive Health, Johns Hopkins Bloomberg School of Public Health, Baltimore, Maryland, United States of America; 2 Department of Community Medicine, College of Medicine, University of Ibadan, Ibadan, Nigeria; 3 Patrick Kayembe Research Center, Kinshasa School of Public Health, Kinshasa, Democratic Republic of the Congo; 4 Institut Supérieur des Sciences de la Population (ISSP/University Joseph Ki-Zerbo), Ouagadougou, Burkina Faso; 5 International Centre for Reproductive Health-Kenya, Nairobi, Kenya; 6 Department of Primary Care, Technical University of Mombasa, Ghent University, Ghent, Belgium; 7 École Nationale Supérieure de Statistique et d’Economie Appliquee (ENSEA) of Abidjan, Abidjan, Côte d’Ivoire; 8 Makerere University, Kampala, Uganda; 9 Soins et Santé Primaire, CESP Centre for Research in Epidemiology and Population Health U1018, Inserm, Villejuif, France; UNC Gillings School of Global Public Health: The University of North Carolina at Chapel Hill Gillings School of Global Public Health, UNITED STATES

## Abstract

Many speculated that COVID-19 would severely restrict the delivery of essential health services, including family planning (FP), but evidence of this impact is limited, partly due to data limitations. We use cross-sectional data collected from regional and national samples of health facilities (n = 2,610) offering FP across seven low- and middle-income countries (LMICs) between 2019 and 2021, with longitudinal data from four geographies, to examine reported disruptions to the FP service environment during COVID-19, assess how these disruptions varied according to health system characteristics, and evaluate how disruptions evolved throughout the first two years of the pandemic, relative to a pre-pandemic period. Findings show significant variation in the impact of COVID-19 on facility-based FP services across LMICs, with the largest disruptions to services occurring in Rajasthan, India, where COVID-19 cases were highest among geographies sampled, while in most sub-Saharan African settings there were limited disruptions impacting FP service availability, method provision, and contraceptive supplies. Facility-reported disruptions to care were not reflected in observed changes to the number of FP clients or types of stockouts experienced in the first two years of the pandemic. Public and higher-level facilities were generally less likely to experience COVID-19-related disruptions to FP services, suggesting policy mitigation measures—particularly those implemented among government-operated health facilities—may have been critical to ensuring sustained delivery of reproductive healthcare during the pandemic.

## Introduction

The onset of the COVID-19 pandemic in March 2020 required governments to reevaluate their health systems and adapt service delivery to a rapidly changing environment. Swift implementation of national and sub-national policies, such as social distancing requirements, stay-at-home orders, public mask mandates, and curfews, dramatically impacted the lives and well-being of people around the globe. These changes and related disruptions to health services were predicted to decrease access to family planning (FP). Experts were concerned that the redistribution of resources toward mitigating the direct effects of the pandemic [1] might inadvertently contribute to increased risk of unintended pregnancy and related maternal morbidity and mortality, particularly in low- and middle-income countries (LMICs) [2]. Early projections estimated that 60 million fewer women would be able to access modern contraception during COVID-19, contributing to 15 million additional unintended pregnancies [2]. Despite bleak projections, data from early in the pandemic showed limited changes in population-level need for contraception and little impact on individuals’ contraceptive use practices [3–6]. Steady levels of contraceptive use provided initial evidence that disruptions to reproductive health services may have been short-lived or small in magnitude or may have reflected successful mitigation strategies, such as reliance on the informal health sector and self-managed contraceptive care, to compensate for disruptions to facility-based services [5].

Several small-scale studies reported disruptions to the continued provision of SRH services across a variety of facilities in LMICs [7–13]. One study conducted in Burkina Faso, Ethiopia, and Nigeria found that 5% of health providers reported a complete halt in family planning services, and 53% reported a partial interruption during the pandemic [7]. Similarly, a qualitative study conducted among Ugandan healthcare providers in 2020 found financial, psychosocial, and mobility barriers impeded access to contraceptive services for providers and clients alike [9].

Other studies showed more positive or mixed findings related to the resilience of health services. An analysis conducted across six countries in 2021, including Kenya, Ethiopia, Zambia, Bangladesh, Indonesia, and Pakistan, found that, despite “ripple effects” of COVID-19 across supply chain functions, many health systems were able to mitigate disruptions to SRH services [14]. Another study quantified these disruptions using health management information systems (HMIS) data across more than 60,000 facilities in eight countries, including Cameroon, Democratic Republic of the Congo (DRC), Liberia, Malawi, Mali, Nigeria, Sierra Leone, and Somalia, identifying family planning consultations between March-July 2020 ranged from 17% lower to 11% higher than expected [15]. HMIS data also suggested rapid post-lockdown service rebounds [16–18], reducing the anticipated negative impact on contraceptive coverage, especially in contexts where more women use long-acting, provider-dependent contraception, such as implants and IUDs. A scoping review found varying declines in the provision of family planning during the first year of the pandemic, with rare occurrences of severe or sustained disruptions, further substantiating these overall trends [19].

While DHIS2 and HMIS data started to illuminate COVID-19’s impact on SRH services [15], concerns about the completeness and quality of these data in many LMICs prior to the pandemic may limit their utility for understanding changes in service delivery environments [20–24]. Additionally, data from private health facilities are insufficiently captured in these sources, resulting in an incomplete or skewed picture of FP services, particularly in settings where the private sector dominates the delivery of contraceptive care, such as DRC and Indonesia [25, 26]. In the context of the pandemic, quality of registry data may have also suffered from staff shortages and shifting responsibilities, thereby limiting the field’s understanding of the pandemic’s effects. Facility records are one essential tool for monitoring provision of services yet fail to capture the broader nature of disruptions related to provider availability or facility closures that shape individuals’ access to care. Documenting reported impacts of COVID-19 from the perspectives of facility leaders and contextualizing these changes alongside longitudinal indicators of contraceptive service availability, method provision, and stockouts before and during the pandemic may elucidate information about the diversity of FP service disruptions.

This study quantifies disruptions to FP services during the first two years of the COVID-19 pandemic using facility-based cross-sectional data from national or regional samples of health facilities in seven LMICs, including longitudinal data from four countries. The objectives of this paper are two-fold; first, we examine reported disruptions to the FP service environment during the COVID-19 pandemic and assess how these disruptions varied according to facility characteristics in seven LMICs; second, we evaluate how disruptions evolved throughout the first two years of the pandemic, relative to a pre-pandemic period, in four countries.

## Methods

### Study procedures and sample

This analysis uses data collected by Performance Monitoring for Action (PMA) in health facilities across seven LMICs, including six countries in sub-Saharan Africa—Kenya, Burkina Faso, DRC (Kinshasa and Kongo Central regions), Nigeria (Lagos and Kano states), Côte d’Ivoire, and Uganda—and one country in south Asia—India (Rajasthan state). These countries or regions were chosen due to their inclusion in the PMA project, which monitors progress towards improving access to family planning and collects repeated facility-based indicators of FP services. In India, PMA only collects data from Rajasthan state, thus, data on family planning service disruptions during COVID-19 are only explored for this state and not at the country-level. Due to COVID-19, PMA had to suspend initial data collection in four countries, Niger, Uganda, Cote d’Ivoire, and India (Rajasthan), thus, longitudinal data from before COVID-19 are not available in these geographies. Across all geographies, cross-sectional data were collected during COVID-19 between August 2020–January 2021. In Burkina Faso, DRC, Kenya, and Nigeria, three phases of longitudinal data were collected, including pre-pandemic (December 2019–January 2020; “baseline”) one year later (November 2020–January 2021; “one-year follow-up”), and two years later (November 2021–February 2022; “two-year follow-up”).

Service delivery points (SDPs) (hereafter termed “facilities”) were selected from a random sample of census-identified geographic areas, selected proportional to size. All public and private facilities serving the selected geographic clusters were listed, including facilities serving the catchment area. Facility sample selection varied by managing authority; private facilities were selected randomly, while public were selected to include the tertiary, secondary, and primary facilities that served the geographic areas selected. In areas with three or fewer private facilities, all were included. The total facility sample included a total of 3,052 facilities across the seven geographies. We restricted our analysis to facilities that offered FP, resulting in a final analytical sample of 2,610 facilities, spanning Burkina Faso (n = 228), Côte d’Ivoire (n = 192), DRC (n = 273), Kenya (n = 904), Nigeria (n = 173), Rajasthan (n = 507), and Uganda (n = 333). Longitudinal analyses exploring changes in FP services from pre-pandemic through the first two years of the pandemic were limited to the facilities where such data were available: Burkina Faso (n = 210), DRC (n = 189), Kenya (n = 859), Nigeria (n = 152).

Trained data collectors in each geography conducted the facility surveys, beginning with informed consent procedures for each facility respondent, such as a facility manager, main administrator, or family planning in-charge, identified to complete the interview. Per health facility and at each time point (if applicable), the data collector administered informed consent and provided a paper copy of the consent form to each facility respondent. Data collectors recorded the facility respondent’s consent as part of the survey via the respondent’s signature or checking a response box to indicate their agreement. Consenting facility respondents provided their responses to a series of questions about FP service delivery and operational challenges experienced during COVID-19. Data on contraceptive method provision within each facility were collected directly from the facility’s family planning register, recorded by the facility’s data supervisor. Surveys lasted approximately 30–75 minutes. Ethical approval for the data collection activities, including informed consent procedures, was provided by in-country review boards, including the Ethics Committee for Health Research at the Ministry of Health and Ministry of Higher Education, Scientific Research and Innovation (Burkina Faso; N/Refs: A018-2019; A14-2020/CEIRES; ISSP/DA/GG/062/2021); Comité National d’Ethique des Sciences de la Vie et de la Santté at the Ministry of Health of Côte d’Ivoire (N/Refs: 053-2-/MSHP/CNESVS-km; 250-21/MSHP/NCESVS-km; 128-22/MSHPCMU/CNESVS-kp); University of Kinshasa School of Public Health (DRC) (N/Refs: ESP/CE/030B/2019; ESP/CE/78/2020; ESP/CE/160/2020; ESP/CE/159B/2021); Kenyatta National Hospital-University of Nairobi Scientific Ethics Review Committee (N/Refs: KHN-ERC/A/412; KHN-ERC/A/150; KHN-ERC/Mod&SAE/172); Lagos State University Teaching Hospital Research Ethics Committee (N/Refs: LREC/06/10/1276) and Kano State Health Research Ethics Committee of the Ministry of Health and the Research Ethics Committee of the Aminu Kano Teaching Hospital in Kano (Nigeria) (N/Refs: MOH/Off/797/TI/1487; MOH/Off/797/TI/2006; MOH/Off/797/TI/2096; SHREC/2021/2880); Indian Institute of Health Management Research University Institutional Committee for Ethics and Review of Research (Rajasthan) (N/Ref: 0990–0279), and Makerere University School of Public Health (Uganda) (N/Ref: HDREC805). The SDP survey was deemed IRB-exempt as non-human subjects research by the Johns Hopkins Bloomberg School of Public Health.

### Study context

The seven countries or regions where this study takes place experienced COVID-19 in diverse ways, including variation in number of Coronavirus cases, the rate of increase in cases over time, mortality, and policy responses to the pandemic. Details on the timing of data collection within the context of COVID-19 impacts and policy changes in each country are provided in Table 1; data specific to Rajasthan state were unavailable, thus, national data are reported for India [27]. Early in the pandemic, data collected between August 2020 and January 2021 indicated that the cumulative number of COVID-19 cases ranged widely across geographies, from 2,931 cases in Burkina Faso to nearly two million cases in India [27]. Policies to mitigate the spread of Coronavirus included face covering requirements in all public spaces (Burkina Faso, DRC, Kenya, and Nigeria) or any time outside the home (Côte d’Ivoire, India, and Uganda), stay-at-home orders (India, Kenya, Nigeria, Uganda), and workplace and school closures in some geographies. Roughly two years into the pandemic, between December 2021 and February 2022, the cumulative number of cases had quadrupled in most geographies with longitudinal data; for example, in Kenya, cases rose from 55,877 to 255,260 and in DRC cases grew from 12,859 to 58,319 during this period [27].

**Table 1:** Facility survey administration schedule by site and COVID-19 context.

							National policy measures to mitigate spread of Coronavirus			
	Site	Survey	Dates of data collection	Cumulative number of confirmed COVID-19 cases*	Cumulative number of confirmed COVID-19 deaths*	COVID-19 Stringency Index* (range: 0–100; 100 = strictest)	Stay-at-home requirement*	Face coverings required*	School closures*	Workplace closures*
**Cross-sectional data only**										
	Côte d’Ivoire	Baseline	Sept ‐ Oct 2020	18,103	126	38.0	Recommended	Required outside the home at all times	Required closures (some levels)	No measures
	India^	Baseline	Aug ‐ Sep 2020	1,800,000	37,367	79.6	Required (except for essentials)	Required outside the home at all times	Required closures (all levels)	Required for some
	Uganda	Baseline	Sep ‐ Oct 2020	3,037	32	76.9	Required (except for essentials)	Required outside the home at all times	Required closures (all levels)	Required for some
**Longitudinal data**										
	Burkina Faso	Baseline	Dec 2019 ‐ Jan 2020	‐‐	‐‐	‐‐	‐‐	‐‐	‐‐	‐‐
		One-year follow-up	Dec 2020 ‐ Jan 2021	2,931	68	13.9	No measures	Required in all public spaces	No measures	No measures
		Two-year follow-up	Dec 2021 ‐ Feb 2022	16,000	286	13.9	Required (except for essentials)	Required in all public spaces	No measures	No measures
	Democratic Republic of the Congo (DRC)	Baseline	Dec 2019 ‐ Jan 2020	‐‐	‐‐	‐‐	‐‐	‐‐	‐‐	‐‐
		One-year follow-up	Dec 2020 ‐ Jan 2021	12,859	335	22.2	No measures	Required in all public spaces	Recommended	Recommended
		Two-year follow-up	Dec 2021 ‐ Feb 2022	58,319	1,107	39.8	No measures	Required in all public spaces	No measures	Recommended
	Kenya	Baseline	Nov ‐ Dec 2019	‐‐	‐‐	‐‐	‐‐	‐‐	‐‐	‐‐
		One-year follow-up	Nov ‐ Dec 2020	55,877	1,013	68.5	Required (except for essentials)	Required in some public spaces	Required closures (some levels)	Recommended
		Two-year follow-up	Nov 2021 ‐ Jan 2022	255,260	5,335	36.1	Recommended	Always required outside the home	No measures	Required for some
	Nigeria	Baseline	Dec 2019 ‐ Jan 2020	‐‐	‐‐	‐‐	‐‐	‐‐	‐‐	‐‐
		One-year follow-up	Dec 2020 ‐ Jan 2021	67,838	1,176	50.9	Required (except for essentials)	Required in all public spaces	Recommended	Recommended
		Two-year follow-up	Dec 2021 ‐ Feb 2022	214,270	2,978	37.9	Required (except for essentials)	Required in all public spaces	No measures	No measures

Note: *by first day of survey administration month. Data from www.https://ourworldindata.org/coronavirus; accessed January 9, 2023.

^Information about COVID-19 in India (cumulative cases, deaths, and policy responses) were only available at national level; data from this study were collected in Rajasthan state.

### Measures

Descriptions of study measures and their definitions are provided in Table 2. We first explored eight indicators of reported COVID-19 disruptions, measured cross-sectionally between August 2020 and January 2021, which were grouped into four categories by disruption type, including challenges to 1) service availability, 2) provider availability, 3) administrative capacity, and 4) contraceptive supplies. First, disruptions to service availability included three indicators: facility closures during COVID-19 (never, <3 weeks, 3 or more weeks); reduced days or hours of operation (yes/no); and suspension of FP services (never, <3 weeks, 3 or more weeks). Second, disruptions to provider availability were measured using three indicators: reassignment of FP providers to COVID-19 duties (yes/no); increased provider absenteeism (yes/no); and among facilities offering provider-dependent methods (i.e., sterilization, implants, IUDs, and injectables), suspension of these methods (yes/no). Third, administrative capacity disruptions were measured as inability to maintain client FP records (yes/no). Fourth, our indicator of contraceptive supply disruptions assessed perceived changes in the supply of family planning commodities during COVID-19 restrictions by asking respondents, “How regular was the supply of family planning methods to this facility during the time of Coronavirus (COVID-19) restrictions?” (no change/regular, more irregular, stopped completely).

**Table 2:** Indicators of COVID-19 disruptions and contraceptive services.

Cross-sectional measures (13 total)				
	**Measure**	**No.**	**Indicator(s) or Definitions**	**Responses or Categorization**
	Service availability	1	Facility closures during COVID-19	Never, <3 weeks, 3 or more weeks
		2	Reduced days or hours of operation	Yes/No
		3	Suspension of family planning services	Never, <3 weeks, 3 or more weeks
	Provider availability	4	Reassignment of FP providers to COVID-19 duties	Yes/No
		5	Increased provider absenteeism	Yes/No
		6	Suspension of provider-dependent methods^	Yes/No
	Administrative capacity	7	Inability to maintain client FP records	Yes/No
	Contraceptive supplies	8	Perceived regularity of family planning method supplies during COVID-19 restrictions	
	Any COVID-19-related disruption	9	Reporting at least one service availability, provider availability, administrative capacity, or contraceptive supply disruption (Indicators 1–8)	Yes/No
	Any service availability disruption	10	Reporting at least one service availability disruption (Indicators 1–3)	Yes/No
	Any provider availability disruption	11	Reporting at least one provider availability disruption (Indicators 4–6)	Yes/No
	Average number of COVID-19-related disruptions	12	Total number of disruptions to service availability, provider availability, administrative capacity, and contraceptive supply (Indicators 1–8)	Additive measure: min = 0, max = 8
	Demand-related changes	13	Reported reduction in FP clients during COVID-19	None, small, moderate, large
**Longitudinal Measures (10 total)**				
	Service availability	1	Number of days in a week FP services/products were offered	Additive measure: min = 0, max = 7
	Method provision	2	Average total number of clients in the last month (sum of number of FP visits for each method offered)	Additive measure
		3	Average total number of provider-dependent clients (i.e., sterilization, implant, IUD, injectables)	Additive measure
		4	Average total number of provider-independent clients (i.e., pills, emergency contraception, condoms, diaphragm, foam)	Additive measure
	Contraceptive stockouts*	5	Stockout of at least one method	Yes/No
		6	Stockout of at least one provider-dependent method	Yes/No
		7	Stockout of at least one provider-independent method	Yes/No
		8	Stockout of at least two of six modern method categories	Yes/No
		9	Stockout of at least one of six modern method categories	Yes/No
		10	Average total number of methods stocked out	Additive measure: min = 0, max = 10

Notes: ^Among facilities that offered any provider-administered methods, including sterilization, implants, IUDs, and injectables.

*Stockouts in last three months.

Next, we generated four summary measures of reported COVID-19 disruptions, including 1) a binary measure of any COVID-19-related disruption (yes/no), and separate, binary measures of any disruption to 2) service availability (yes/no), or 3) provider availability (yes/no). Our fourth summary measure included an additive indicator of the total number of disruptions, including those affecting service and provider availability, administrative capacity, and contraceptive supply, reported at each facility (min = 0, max = 8) to explore how severity of disruptions varied across contexts. Finally, we assessed reported perceived demand-related changes in FP services by asking, “During the time of Coronavirus (COVID-19) restrictions, did your facility experience any reduction in the number of family planning clients (or purchase of contraceptive products) compared to your usual client numbers?” (no reduction, small reduction, moderate reduction, large reduction).

Using longitudinal data collected in four geographies (Burkina Faso, DRC, Kenya, Nigeria), we also assessed observed changes in contraceptive service environments one year and two years into the pandemic by comparing 10 indicators of service availability, method provision, and contraceptive stockouts across three survey periods. For longitudinal assessment, service availability was operationalized as a continuous variable reflecting the number of days in a week FP services/products were offered (min = 0, max = 7). Method provision was first measured as the total client volume in the last month (number of FP visits for each method offered), recorded from the facility’s FP register and operationalized as a continuous variable. We further explored method provision by grouping contraceptive client volumes into two categories based on method characteristics: total number of clients receiving provider-dependent methods (sterilization, implant, IUDs, injectables) and total number of clients receiving provider-independent methods (pills, EC, condoms, diaphragm, foam).

Contraceptive stockouts in the last three months were assessed through six indicators. First, we used three crude measures, including facility-level stockouts of at least one method; at least one provider-dependent method; and at least one provider-independent method. We then quantified the severity of contraceptive stockouts by summing the total number of methods out of stock in the last three months per facility (min = 0, max = 10; sterilization not assessed, injectables measured as Sayana Press and Depo Provera). Next, we used two measures of modern contraceptive method availability developed by the Reproductive Health Supplies Coalition [28] and recommended by Barden-O’Fallon and Ijdi (2023), including the proportion of facilities “providing at least one modern contraceptive method for at least four of the six method categories available on the day of the assessment: barrier (condoms and spermicide), hormonal short-acting (pill), hormonal medium-acting (injectable), long-acting reversible (implant and IUD), permanent method (male or female sterilization), emergency contraception” and the proportion of facilities “providing at least one modern contraceptive method for each of the six method categories available on the day of assessment” [29]. We examined the complement of these indicators to align with our exploration of method stockouts in the context of COVID-19 disruptions, resulting in two indicators reflecting the proportion of facilities with 1) “stockouts of at least two of six modern contraceptive method categories available on the day of the assessment”, and 2) “stockouts of at least one of six modern contraceptive method categories available on the day of the assessment”. We required all methods to be observed on the day of interview to categorize them as “available”, except for male or female sterilization, which we assessed according to its reported availability as a family planning method offered by the facility.

We also measured several facility characteristics to describe the broader service delivery environment within each geography, including managing authority (public, private), facility type (hospital, health center/clinic, pharmacy/drug shop/other), residence of enumeration areas served by the facility (rural/urban), availability of electricity and water, availability of services to adolescents aged 10–19, and integration of community health volunteers within facilities.

### Analytic methods

Descriptive statistics were used to examine facility characteristics among the sample of facilities participating in the first COVID-19 survey conducted in each geography (i.e., baseline surveys in Côte d’Ivoire, Rajasthan, and Uganda and one-year follow-up surveys in Burkina Faso, DRC, Kenya, and Nigeria) and assess the proportion of facilities reporting each type of COVID-19-related service disruption or demand-related change. Pearson’s chi-squared statistics were used to calculate p-values and evaluate if variations in COVID-19 disruptions differed according to facility characteristics. Statistical significance was set at p<0.05.

Next, using three phases of longitudinal data from Burkina Faso, DRC, Kenya, and Nigeria, we explored changes in the family planning service environment. We first examined indicators of contraceptive service availability and method provision by comparing the average number of days family planning services were offered and total number of family planning clients in the last month, respectively, at each survey; linear regression was used to assess if differences in mean values were statistically significant between surveys (i.e., baseline vs. one-year follow-up, and one-year vs. two-year follow-up). Similarly, we evaluated contraceptive stockout indicators by comparing the proportion of facilities reporting each type of stockout throughout the two-year period; statistical significance of differences in proportions between surveys was assessed through logistic regression. Analyses were site-specific, clustered by facility, and conducted in Stata 16.

## Results

Facility characteristics, by country, are presented in Table 3. Most facilities were public, ranging from 68.8% in Nigeria to 89.6% in Côte d’Ivoire, except for DRC and Rajasthan, where the majority were private (68.1% and 55.6%, respectively). Facility types varied across geographies, though a large proportion were health centers or clinics (e.g., 84.1% in Burkina Faso, 67.3% in Uganda); in Kenya, most facilities (62.7%) were pharmacies, drug shops, or other. Facilities in Kenya, Rajasthan, and Uganda predominantly served rural enumeration areas, while the opposite was true in other geographies. Challenges to infrastructure were most common in DRC, where more than half of facilities reported outages of electricity (59.2%) and water (55.3%) for more than two of the past 24 hours; similar levels were observed in Nigeria (47.5% and 44.4%, respectively). Facility integration of community health volunteers ranged from 13.1% in Côte d’Ivoire to 64.4% in Kenya. Nearly all facilities offered family planning services to adolescents.

**Table 3:** Characteristics of facilities offering family planning services, by geography %(n).

		Burkina Faso	Côte d’Ivoire	DRC	Kenya	Nigeria	Rajasthan	Uganda
		(n = 228)	(n = 192)	(n = 273)	(n = 904)	(n = 173)	(N = 507)	(n = 333)
Managing authority								
	Public	193 (84.7)	172 (89.6)	87 (31.9)	774 (85.6)	119 (68.8)	225 (44.4)	257 (77.2)
	Private	35 (15.4)	20 (10.4)	186 (68.1)	130 (14.4)	54 (31.2)	282 (55.6)	76 (22.8)
Facility type								
	Hospital	14 (6.1)	67 (34.9)	46 (16.9)	98 (10.8)	42 (24.3)	41 (8.1)	52 (15.6)
	Health center/clinic	192 (84.1)	99 (51.6)	131 (48.0)	239 (26.4)	74 (42.8)	265 (52.3)	224 (67.3)
	Pharmacy/Drug shop/Other	22 (9.7)	26 (13.5)	96 (35.2)	567 (62.7)	57 (33.0)	201 (39.6)	57 (17.1)
Residence of EA served								
	Urban	137 (63.7)	99 (51.6)	273 (100.0)	315 (36.5)	133 (79.2)	206 (40.6)	120 (36.0)
	Rural	78 (36.3)	93 (48.4)	‐‐	548 (63.5)	35 (20.4)	301 (59.4)	213 (64.0)
Infrastructure								
	Electricity outages*	20 (8.8)	19 (9.9)	162 (59.3)	222 (24.6)	84 (48.6)	101(19.9)	102 (30.6)
	Water outages*	43 (18.9)	75 (39.1)	151 (55.3)	253 (28.0)	80 (46.2)	214 (42.4)	123 (36.9)
Average days FP offered, mean(sd)		6.8 (0.8)	5.9 (1.2)	5.9 (2.0)	5.3 (0.9)	5.3 (1.5)	6.8 (0.9)	5.5 (1.4)
Offers adolescent family planning^		225 (98.7)	19 (99.5)	243 (89.3)	884 (97.8)	155 (89.6)	445 (88.1)	322 (96.7)
Facility integration of community health volunteers (CHVs)		44 (19.3)	26 (13.5)	84 (30.8)	576 (63.7)	51 (29.5)	183 (36.1)	196 (58.9)

Notes: *Facility experienced an outage of electricity/water for more than two hours in past 24 hours. EA = Enumeration Area served by the facility. p-val = p-value from chi-squared test.

^Adolescent family planning: facility offers family planning services to adolescents aged 10–19.

### Reported changes in the FP service environment during COVID-19

COVID-19’s impact on FP services ranged widely across geographies (Table 4). Overall, reports of FP service disruptions were highest in Rajasthan across nearly all indicators, followed by Nigeria and Uganda. Several sub-Saharan African countries, including Côte d’Ivoire, Burkina Faso, and DRC, reported relatively minimal effect of COVID-19 in terms of FP service availability, with fewer than 5% of facilities reporting closures or suspended services. While most facilities across geographies reported FP services remained available, a considerable proportion—including one-quarter of facilities in DRC and Uganda and nearly half in Rajasthan—experienced reduced hours of operation. Facility closures were rare, although 22.9% of facilities in Rajasthan and approximately 5% of facilities in Nigeria and Uganda suspended FP services completely for three weeks or more at some point during the pandemic.

**Table 4:** Among facilities offering family planning (FP), the proportion (%) reporting disruptions to FP services during COVID-19, by geography.

Type of COVID-19 disruption		Burkina Faso (n = 228)	Côte d’Ivoire (n = 192)	DRC (n = 273)	Kenya (n = 904)	Nigeria (n = 173)	Rajasthan (n = 507)	Uganda (n = 333)
**Service availability**								
Facility closed								
	Never	97.8	99.0	98.6	96.8	88.4	72.5	94.3
	<3 weeks	1.8	0.5	0.7	2.0	4.6	6.3	1.2
	3 weeks or more	0.4	0.5	0.7	1.2	7.0	21.2	4.5
Reduced hours/days of service		5.3	5.7	23.4	15.5	36.4	44.0	26.1
Suspension of FP services								
	Never	96.5	98.4	98.9	96.9	94.2	74.7	94.6
	<3 weeks	1.8	0.0	0.0	1.3	1.2	2.4	0.9
	3 weeks or more	1.8	1.6	1.1	1.8	4.6	22.9	4.5
**Provider availability**								
Reassignment of FP providers		13.7	8.9	11.0	12.1	16.3	36.4	15.0
High provider absenteeism		4.0	2.6	7.7	11.0	29.7	18.2	26.7
Suspension of provider-dependent methods^		3.4	4.8	5.9	5.1	6.9	31.3	7.9
**Administrative capacity**								
Inability to maintain FP records		9.7	9.9	27.8	12.1	20.8	40.2	25.2
**Contraceptive supplies**								
Regularity of FP supplies								
	No change/regular	89.4	82.1	78.2	77.4	75.4	62.8	76.1
	More irregular	9.7	16.3	18.4	21.5	21.1	22.0	20.3
	Stopped completely	0.9	1.6	3.4	1.1	3.5	15.3	3.6
Any COVID-19 disruption*		32.9	33.9	60.1	52.7	69.9	87.4	61.3
Average number of COVID-19 disruptions, mean(sd)		0.5 (0.9)	0.5 (0.8)	0.9 (1.0)	0.8 (1.0)	1.4 (1.4)	2.3 (1.8)	1.3 (1.4)
**Demand-related changes**								
Reduction in FP clients during COVID-19								
	None	69.9	45.3	31.5	36.1	27.9	21.5	33.7
	Small	19.9	31.8	37.0	31.1	33.1	12.7	25.6
	Moderate	9.4	11.5	13.0	24.6	15.7	42.3	24.4
	Large	1.8	11.5	18.5	8.3	23.3	23.5	16.3

Notes: FP = family planning.

^Among facilities that offered at least one provider-dependent method, including sterilization, implants, IUDs, and injectables.

*Any COVID-19 disruption to family planning services includes facilities reporting at least one of the following: closure for any duration, reduced hours or days of service, suspension of FP services, reassignment of FP providers, high absenteeism of providers, more irregular or complete stop of FP supplies; does not include demand-related changes.

Challenges to provider availability, including provider reassignment and absenteeism were common across contexts. Reassignment of FP providers to COVID-19-related duties ranged from 8.9% to 15.0% in sub-Saharan countries, rising to 36.4% in Rajasthan, while high absenteeism of providers was most reported in Uganda and Nigeria (26.7% and 29.7%, respectively). Among facilities offering provider-administered methods, including sterilization, IUDs, implants, and injectables, suspension of these methods as part of FP services was most common in Rajasthan—reported by nearly one-third of facilities (31.3%)—followed by Nigeria and Uganda (6.9% and 7.9%, respectively). Administrative capacity was limited with many facilities indicating they were unable to maintain client FP records during COVID-19, ranging from 9.7% of facilities in Burkina Faso to 40.2% in Rajasthan. Finally, while most facilities reported no change in the regularity of contraceptive supplies, 15.3% in Rajasthan experienced a complete stop in supply, and one in five facilities in Kenya, Uganda, Nigeria, and Rajasthan reported supply chain irregularities during COVID-19.

Most facilities, except for those in Burkina Faso and Côte d’Ivoire, experienced at least one COVID-19-related service disruption, ranging from 60.1% in DRC to 87.4% in Rajasthan, and resulting in the highest average of 2.3 disruptions per facility in Rajasthan. Reported demand-related changes induced by COVID-19 restrictions varied considerably across sites. While most facilities in Burkina Faso reported experiencing no reduction in FP clients (69.9%), a nearly equal proportion in Rajasthan reported moderate (42.3%) or large (23.5%) declines in client volumes—an experience shared by roughly 30–40% of facilities in DRC, Kenya, Nigeria, and Uganda.

### Variation to the health service environment according to facility characteristics

Experience of any COVID-19-related disruptions varied by facility characteristics in all geographies except Nigeria and DRC (Table 5). A greater proportion of private facilities reported at least one disruption to service availability, administrative capacity, or contraceptive supplies in Burkina Faso, Côte d’Ivoire, Kenya, and Uganda, compared to public facilities. In Rajasthan and Uganda, more non-hospital facilities (i.e., health centers, clinics, and pharmacies/drug shops) reported at least one disruption, relative to hospitals, while fewer health centers/clinics were impacted in Burkina Faso. Facilities serving urban areas in Burkina Faso and Uganda were also more likely to experience at least one disruption.

**Table 5:** Among facilities offering family planning (FP), the proportion of facilities that experienced each type of COVID-19-related disruptions to FP services, by disruption type, facility characteristics, and geography.

		Burkina Faso (n = 228)		Côte d’Ivoire (n = 192)		DRC (n = 273)		Kenya (n = 904)		Nigeria (n = 173)		Rajasthan (n = 507)		Uganda (n = 333)	
		**Any COVID-19-related disruption to service or provider availability, administrative capacity, or contraceptive supplies**													
		%	p-val	%	p-val	%	p-val	%	p-val	%	p-val	%	p-val	%	p-val
**Managing authority**															
	Public	26.9	**<0.001**	31.4	**0.035**	57.5	0.548	47.9	**<0.001**	66.4	0.130	88.4	0.518	50.6	**<0.001**
	Private	65.7		55.0		61.3		80.8		77.8		86.5		97.3	
**Facility type**															
	Hospital	35.7	**0.020**	28.4	0.139	60.9	0.911	46.9	0.166	69.1	0.160	68.3	**<0.001**	50.0	**<0.001**
	Health center/clinic	29.7		33.3		61.1		57.3		63.5		92.8		54.9	
	Pharmacy/Drug shop+	59.1		50.0		58.3		51.7		79.0		84.2		96.5	
**Residence of EA served**															
	Urban	40.9	**0.002**	38.4	0.171	‐‐	‐‐	56.5	0.065	66.9	0.066	84.0	0.057	70.8	**0.007**
	Rural	20.5		29.0				50.0		82.9		89.7		55.8	
		**Any service availability disruption**													
		%	p-val	%	p-val	%	p-val	%	p-val	%	p-val	%	p-val	%	p-val
**Managing authority**															
	Public	3.1	**<0.001**	4.1	**<0.001**	39.1	**<0.001**	13.1	**<0.001**	34.5	**0.030**	26.7	**<0.001**	9.7	**<0.001**
	Private	34.3		25.0		18.8		52.3		51.9		74.1		86.8	
**Facility type**															
	Hospital	7.1	**<0.001**	9.0	**0.002**	28.3	**<0.001**	16.3	0.775	31.0	**0.009**	22.0	**<0.001**	11.5	**<0.001**
	Health center/clinic	5.2		1.0		35.1		19.7		32.4		45.6		15.2	
	Pharmacy/Drug shop+	31.8		19.2		10.4		18.7		56.1		67.0		89.5	
**Residence of EA served**															
	Urban	10.2	0.195	8.1	0.280	‐‐	‐‐	27.9	**<0.001**	38.4	0.428	55.8	0.302	36.7	**0.004**
	Rural	5.1		4.3				13.3		45.7		51.2		22.1	
		**Any provider availability disruption**													
		%	p-val	%	p-val	%	p-val	%	p-val	%	p-val	%	p-val	%	p-val
**Managing authority**															
	Public	19.2	0.272	15.7	0.501	20.7	0.878	22.2	0.426	42.9	0.341	85.3	**<0.001**	38.1	0.833
	Private	11.4		10.0		19.9		25.4		35.2		28.7		39.5	
**Facility type**															
	Hospital	28.6	**0.047**	11.9	0.470	28.3	**0.025**	25.5	0.622	45.2	0.248	2.3	**<0.001**	36.5	0.608
	Health center/clinic	19.3		18.2		23.7		23.9		44.6		76.8		40.2	
	Pharmacy/Drug shop+	0.0		11.5		11.5		21.7		31.6		21.2		33.3	
**Residence of EA served**															
	Urban	24.1	0.006	19.2	0.103	‐‐	‐‐	26.4	**0.020**	42.9	0.220	43.2	**<0.001**	49.2	**0.003**
	Rural	9.0		10.8				19.5		31.4		61.1		32.4	
		**Disruption to administrative capacity (i.e., FP record-keeping)**													
		%	p-val	%	p-val	%	p-val	%	p-val	%	p-val	%	p-val	%	p-val
**Managing authority**															
	Public	5.2	**<0.001**	6.4	**<0.001**	6.9	**<0.001**	8.9	**<0.001**	9.2	**<0.001**	18.2	**<0.001**	10.9	**<0.001**
	Private	34.3		40.0		37.6		30.8		46.3		57.8		73.7	
**Facility type**															
	Hospital	7.1	**<0.001**	6.0	**0.001**	10.9	**<0.001**	6.1	0.115	16.7	**<0.001**	12.2	**<0.001**	9.6	**<0.001**
	Health center/clinic	6.3		7.1		21.4		14.2		9.5		37.3		15.6	
	Pharmacy/Drug shop+	40.9		30.8		44.8		12.2		38.6		49.8		77.2	
**Residence of EA served**															
	Urban	11.0	0.439	9.1	0.700	‐‐	‐‐	14.6	**0.045**	20.3	0.969	38.4	0.473	20.8	0.166
	Rural	7.7		10.8				10.0		20.0		41.5		27.7	
		**Disruption to regularity of contraceptive supplies**													
		%	p-val	%	p-val	%	p-val	%	p-val	%	p-val	%	p-val	%	p-val
**Managing authority**															
	Public	9.3	0.166	18.0	0.737	16.1	0.155	20.2	**<0.001**	21.0	0.137	19.1	**<0.001**	13.6	**<0.001**
	Private	17.4		15.0		23.7		36.9		31.5		51.4		57.9	
**Facility type**															
	Hospital	7.1	0.445	13.4	0.413	19.6	0.928	20.4	0.834	33.3	**<0.001**	14.6	**0.001**	9.6	**<0.001**
	Health center/clinic	9.9		21.2		22.1		23.4		9.5		35.4		17.9	
	Pharmacy/Drug shop+	18.2		15.4		20.8		22.6		36.8		43.8		59.7	
**Residence of EA served**															
	Urban	13.1	0.125	19.2	0.578	‐‐	‐‐	19.7	0.107	18.1	**<0.001**	36.9	0.942	25.0	0.681
	Rural	6.4		16.1				24.5		48.6		37.2		23.0	

Notes: EA = Enumeration Area served by the facility. p-val = p-value from chi-squared test comparing outcomes by facility characteristics. +Includes “Other” facility types.

These patterns remained when we examined differences by disruption type. Private health facilities were more likely to report any service availability or administrative capacity disruption across nearly all geographies and were also more likely to experience a contraceptive supply disruption in Kenya, Rajasthan, and Uganda. Rajasthan was the only geography where provider availability disruptions varied by managing authority and were reported by 85.3% public facilities, relative to 28.7% of private facilities. Pharmacies/drug shops were also disproportionately affected by disruptions to service availability, except in DRC where health centers/clinics were more impacted, and in Kenya where such disruptions did not vary by facility type. Similarly, these facilities were most affected by administrative capacity disruptions in all geographies except Kenya and regularity of contraceptive supplies in Nigeria, Rajasthan, and Uganda. In contrast, hospitals in Burkina Faso and DRC and health centers/clinics in Rajasthan were more likely to report any disruption to provider availability. While fewer rural facilities were affected by service and provider availability disruptions in Kenya and Uganda, they were more affected by disruptions to contraceptive supplies in Nigeria and to provider availability in Rajasthan.

### Longitudinal changes in family planning services

**Fig 1:**
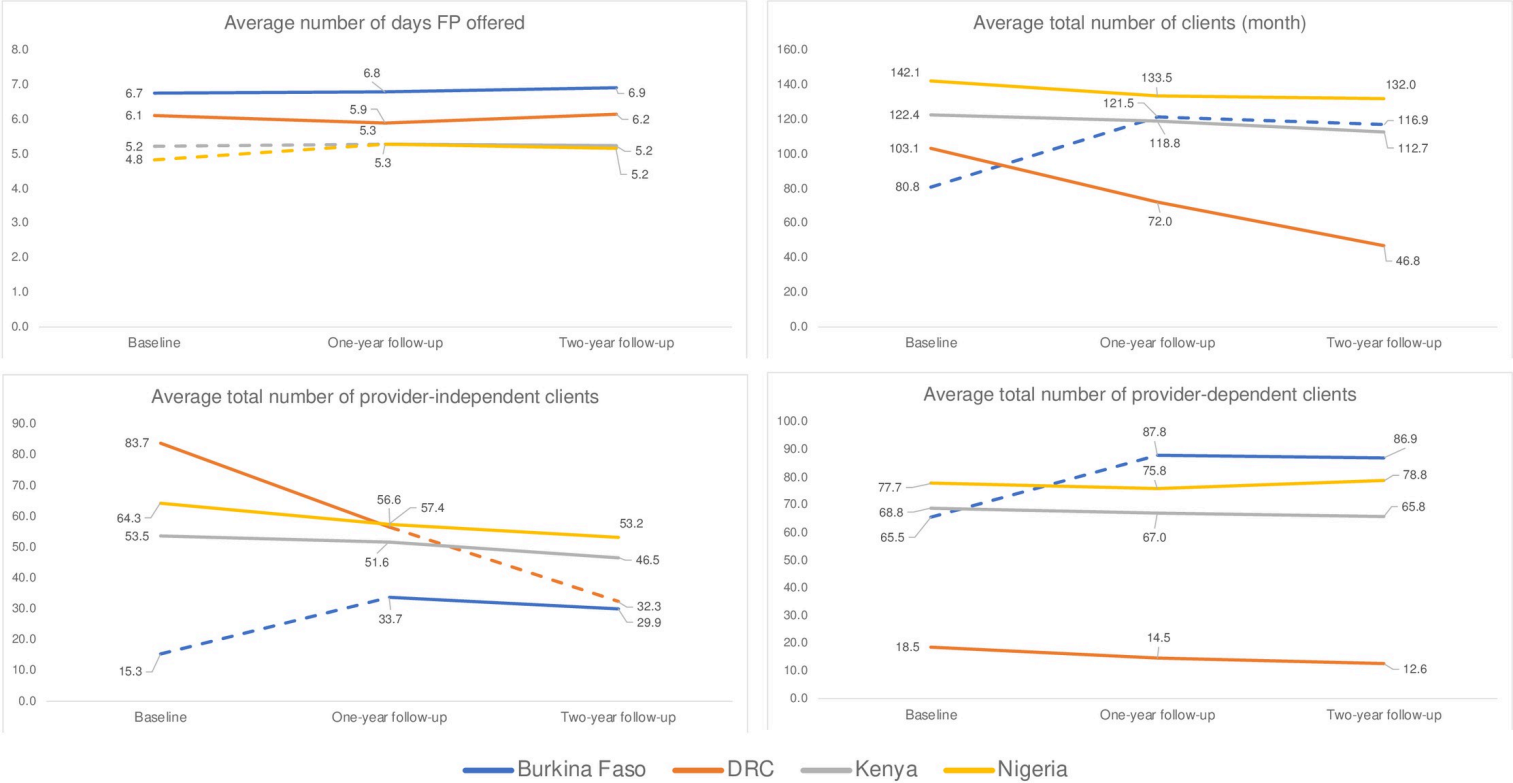
Contraceptive service availability and method provision indicators, by geography*. *Dashed lines indicate statistically significant change in indicator between time points at the p<0.05 level. DRC = Democratic Republic of Congo; FP = family planning.

Fig 1 illustrates changes in contraceptive service availability and method provision by geography with statistically significant changes in indicators presented as dashed lines. Between baseline (pre-pandemic; 2019) and follow-up surveys one year later (2020), the average number of days family planning services were offered remained stable and even increased slightly in Kenya and Nigeria (rising from 5.2 to 5.3 days and 4.8 to 5.3 days, respectively; p<0.05). No significant differences were observed between the one- and two-year follow-up surveys later in the pandemic. Changes to the provision of family planning methods were modest and mostly non-significant, except for an increase in the average number of past-month clients in Burkina Faso, which rose from 80.8 to 121.5 clients between late 2019 and 2020 and declined slightly (116.9 clients) by 2021 (p<0.001).

Method-specific provision reflected these fluctuations, though changes were modest and varied. In Burkina Faso, increases in the number of family planning clients were attributable to a combination of additional users of provider-dependent and provider-independent methods, which increased from 65.5 to 87.8 clients and 15.3 clients to 33.7 clients, respectively, between 2019 and 2020 (p<0.001). In DRC, statistically significant declines in the provision of provider-independent methods were observed between 2020 and 2021, dropping from an average of 57.4 to 32.3 clients. Facilities in Kenya and Nigeria reported no significant changes in method provision, even when exploring variability by method type.

**Fig 2:**
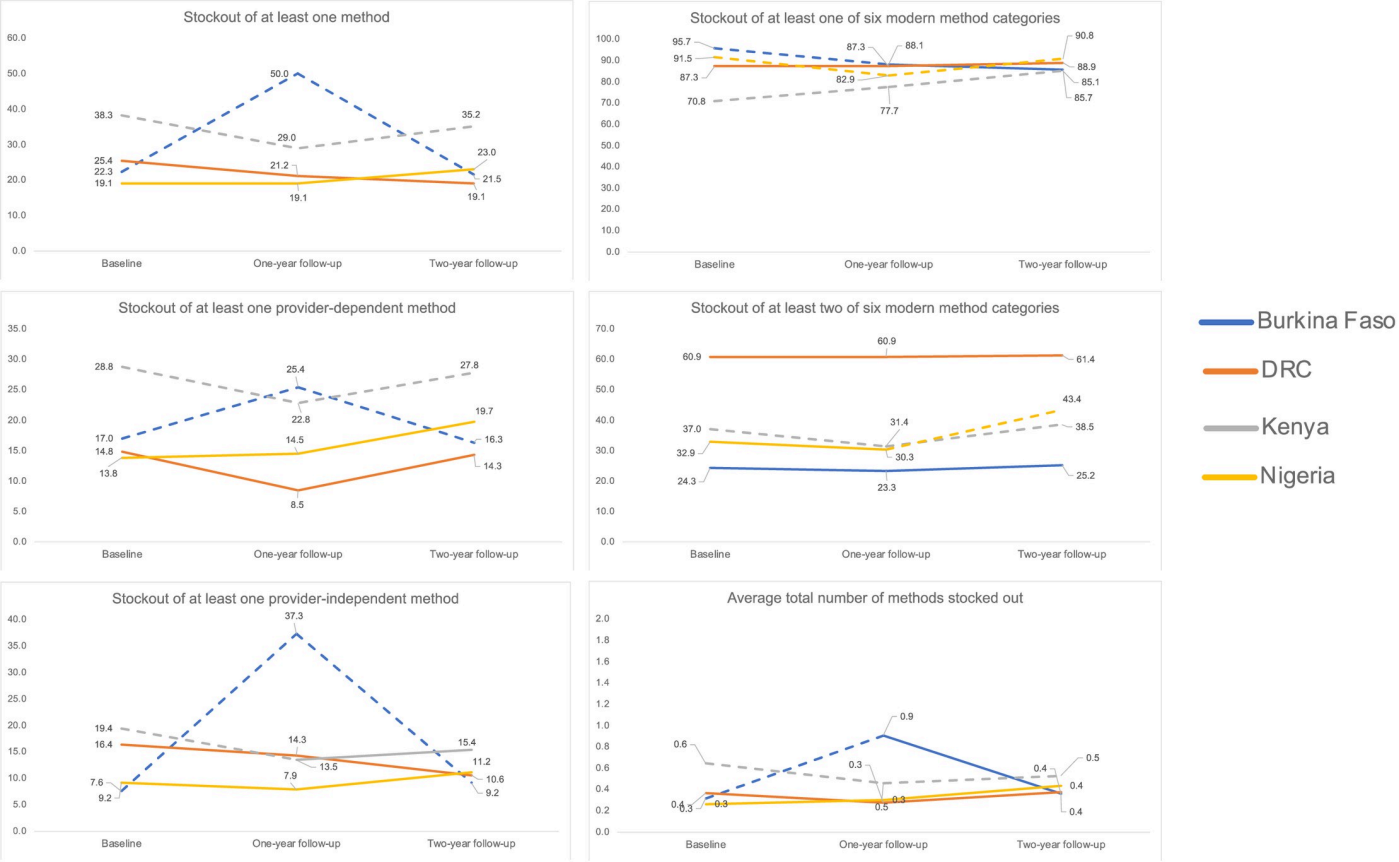
Contraceptive stockout indicators, by geography*. *Dashed lines indicate statistically significant change in indicator between time points at the p<0.05 level. DRC = Democratic Republic of Congo; FP = family planning.

Fig 2 presents changes in contraceptive stockouts by geography. Method stockouts increased by the largest margin in Burkina Faso, where half of facilities reported at least one method out of stock in the past three months in 2020, relative to 22.3% in 2019, though recovery to pre-pandemic levels (21.5%) was observed by 2021. A reverse trend was found in Kenya, where any method stockout declined between 2019 and 2020 (38.3% to 29.0%) but increased by 2021 (35.2%). Similar patterns were observed for stockouts of at least one provider-dependent method and stockouts of at least one provider-independent method in both geographies. While no significant changes were identified for these indicators in DRC and Nigeria, exploration of Reproductive Health Supplies Coalition (RHSC)’s comprehensive indicators of method availability revealed a rise in stockouts of at least two of six modern method categories in Kenya and Nigeria between 2020 and 2021 (31.4% to 38.5% and 30.3% to 43.4%, respectively), following a decline in such stockouts in Kenya between 2019 and 2020. Stockouts of at least one of six modern method categories were common, affecting greater than 70% of facilities across geographies and time points and rising in Kenya and Nigeria between 2019 and 2020. Finally, the average total number of methods stocked out was low—at less than one method per facility—throughout the three-year period; a rise and decline pattern was observed in Burkina Faso and a sustained decline in Kenya.

## Discussion

Our multi-country study, leveraging cross-sectional and longitudinal data collected before and during COVID-19, reveals significant variation in the pandemic’s impact on facility-based family planning services in six sub-Saharan African geographies and Rajasthan state in India. We found low-to-moderate reported changes to FP service and provider availability, administrative capacity, and contraceptive supplies across facilities in sub-Saharan Africa, large disruptions to care in Rajasthan, and variability in the experience of disruptions by facility type, with private, non-hospital facilities and those serving urban areas disproportionately affected. Longitudinal results also indicated, however, that key indicators of contraceptive services, method provision, and stockouts were largely stable when compared to the pre-pandemic period. Even in the absence of a pandemic, contraceptive stocks fluctuate considerably, varying by method and health sector, including in several of the geographies studied [30]. Exceptions to the relative stability observed during the pandemic include stockouts that increased in Burkina Faso, declined initially in Kenya but rebounded a year later, and those that rose in Nigeria between one and two years into the pandemic. Overall, findings suggest the severity of COVID-19 translated into a differential impact on FP services. Geographies with greater proliferation of Coronavirus, like Rajasthan, experienced larger disruptions to care, while contexts where cases remained low proved more resilient in sustaining access to FP services.

Low levels of disruptions to service availability overall indicate greater facility readiness to provide FP during the pandemic than initially feared. These findings echo evidence demonstrating the resilience of primary health facilities in eight LMICs, including Bangladesh, Burkina Faso, Chad, Guatemala, Guinea, Liberia, Malawi and Nigeria, which sustained pre-pandemic client volumes despite changes to supplies and staffing during this period [31]. In the case of FP, severe restrictions, including closure of facilities and temporary suspension of services, were uncommon across the sub-Saharan African service environments explored in our study. Key policy changes implemented early in the pandemic may have played a critical role to protecting access to care. In Kenya, Mozambique, Uganda, and Zimbabwe, for example, FP was identified as an essential service within months of the pandemic’s onset, and governments issued guidance for ensuring care could be maintained safely [11]. Innovative and responsive policies implemented at the sub-national level, including the provision of private home-to-facility transport services, meals, and accommodation to health workers in geographies like Lagos, Nigeria [32], facilitated continuity of SRH care amid the pandemic’s myriad challenges to the health system. While the timing of COVID-19 policy developments varied across geographies, most policies reflected the adoption of WHO’s recommendations for continuing essential SRH care amid the pandemic and, therefore, sustained FP access, even if service schedules, contraceptive supplies, and staffing availability were partially impacted. Additionally, diminished demand for SRH services throughout the early pandemic period, particularly due to fears of acquiring coronavirus at health facilities [4, 33–35], may have contributed to the ability of FP services that were still operational to remain so, during this time. In other words, a lower number of clients seeking FP care in some geographies, as reported by other studies, may have translated into the relative low reporting of COVID-19- related disruptions to FP services.

Trends in facility disruptions to FP services echo patterns of limited changes to contraceptive use patterns at the population-level in sub-Saharan Africa during COVID-19. In Burkina Faso, where we identified a rise in the average number of contraceptive clients overall and for both provider-dependent and provider-independent methods, population-level data suggest a similar story; more than one-quarter of women at risk of unintended pregnancy adopted contraception, including provider-dependent methods, in the first six months of the pandemic [4, 6] and sustained contraceptive use was observed later into the pandemic [5]. Parallel findings of COVID-19’s limited impact on client volumes, and even increased contraceptive provision during the early COVID-19 period, have been documented in other sub-Saharan African contexts, including Kenya [36] and South Africa [37]. In Burkina Faso, these findings likely reflect the enactment of national no-cost family planning program in July 2020, which supported a growing number of individuals to access and use FP services. Our results, however, differ from patterns of rapid decline and recovery in FP clients documented in other facility-based studies, such as in Uganda [38], Ethiopia [12], and Ghana [39]. While these differences may be partially attributable to diverse study designs and timing of data collection throughout the pandemic, the overall narrative of resilient health systems—and FP services, specifically—during COVID-19 remains clear. Our study results reinforce the significant impact that decades of sustained investment in FP services have had on FP health systems, including through the strengthening supply chain systems, critical facility infrastructure, and health provider trainings, particularly throughout LMICs.

Findings also elucidated significant disparities in COVID-19’s impact on contraceptive services according to facility characteristics. Public facilities and hospitals were generally more resilient in their sustained delivery of FP services; across nearly all geographies, these facilities were less likely to report COVID-19-related disruptions to FP service availability (e.g., facility closures, suspension of FP care, reduced hours of operation) and administrative capacity (i.e., record-keeping for FP clients), relative to private and lower-level health facilities, such as health centers, clinics, and pharmacies or drug shops. Similarly, public facilities reported lower levels of contraceptive supply disruptions in Kenya, Rajasthan, and Uganda, while this was also the case for hospitals in Nigeria, Rajasthan, and Uganda. Disruptions to provider availability (e.g., reassignment of providers to COVID-19 care, increased absenteeism) were more common in higher level facilities, such as hospitals, throughout Burkina Faso and DRC, and among health centers/clinics in Rajasthan. These disruptions were also more frequently reported by facilities serving rural areas in Kenya and Uganda and among those serving urban areas in Rajasthan. Inequitable impacts of COVID-19 on the FP service environment based on where facilities are located, the scope of services they provide, and how they are managed underscores how varied the pandemic’s effect was between urban and rural populations, but also how policies affecting government-operated facilities may have shielded them against severe disruptions to FP care. Further research is needed to understand how challenges to sustained delivery of FP services were disproportionately experienced among private, non-hospital facilities operating in predominately urban areas and ways these disruptions may be averted in the future to protect access to care.

Our study is not without limitations, including use of data collected at one time point during COVID-19 for understanding reported disruptions to care early in the pandemic. The first cases of Coronavirus were identified at varying times across study geographies, thus, our estimates of reported changes since the onset of COVID-19 restrictions (e.g., decreased FP services, reassignment, and absenteeism of FP providers) reflect different durations of the pandemic’s impact on health services, all captured within a five-month period. The timing of these data may fail to capture acute, but severe, disruptions to FP services experienced at the peak of the pandemic within each geography, thereby underestimating COVID-19’s impact on access to care. We were also unable to assess the quality of facility register data from which our client volumes were calculated. Given that 10–40% of facilities reported inability to maintain client FP records during COVID-19, it is possible that facility FP registers were also impacted, potentially resulting in overestimates of declines in average client volumes. The fact that limited declines in clients—and even increases—were observed, however, suggests this change in documentation did not impact our results and that our findings may depict a greater impact of the pandemic on FP services than was experienced.

Despite these limitations, however, our study has several strengths. Our data reflect large national and regional samples of health facilities from seven LMICs, generating evidence to reflect the FP service environments during COVID-19 in these geographies. Additionally, we investigated changes in FP services using direct reports from facility leaders whose perspectives offer invaluable insight into disruptions to care yet are often excluded from research relying solely on service statistics, such as DHIS2 or HMIS. Longitudinal data available in four countries facilitated comparisons over a three-year period, allowing us to contextualize observed changes to service availability, method provision, and commodity stockouts within the broader FP service environment and examine rebounds to pre-pandemic levels for some indicators.

## Conclusion

Disruptions to the delivery of FP services during the first two years of the COVID-19 pandemic were common among facilities in six countries or regions across sub-Saharan Africa and Rajasthan, India, yet varied in magnitude, duration, and across facility characteristics. Reported disruptions to care at the facility-level were not mirrored by declines to the number of individuals receiving FP services or types of contraceptive stockouts experienced, suggesting minimal negative impacts of COVID-19 on contraceptive access and services. In geographies where Coronavirus cases grew rapidly, however, such as Rajasthan, increased disruptions to FP reflected unintended consequences of shifting priorities for health services and providers. Greater contraceptive stockouts observed among private facilities during this time highlights opportunities for future interventions to strengthen cross-sectoral supply chain management and facilitate sustained access to care. On the whole, decades of investment in FP programs likely played a key role in improving core functions of health systems, helping to buffer the pandemic’s impact on contraceptive access and use. Policies to protect access to essential SRH during COVID-19—for example, the early establishment of FP as an essential service and increased resources for health providers from the federal government and ministries of health—may have proved critical to ensuring continuity of FP care and are likely to have supported women and couples in achieving their reproductive goals.

## Supporting information

S1 Checklist Inclusivity in global research. (DOCX)

## References

[1] Tang K, Gaoshan J, Ahonsi B. Sexual and reproductive health (SRH): a key issue in the emergency response to the coronavirus disease (COVID- 19) outbreak. Reprod Health. 2020;17: 59. doi: 10.1186/s12978-020-0900-9 32326943 PMC7179791

[2] Riley Sully, Ahmed Biddlecom. Estimates of the Potential Impact of the COVID-19 Pandemic on Sexual and Reproductive Health In Low- and Middle-Income Countries. International Perspectives on Sexual and Reproductive Health. 2020;46: 73. doi: 10.1363/46e9020 32343244

[3] Backhaus A. Pregnancies and contraceptive use in four African countries during the COVID-19 pandemic. Populationyearbook. 2022;20. doi: 10.1553/populationyearbook2022.dat.4

[4] Karp C, Wood SN, Guiella G, Gichangi P, Bell SO, Anglewicz P, et al. Contraceptive dynamics during COVID-19 in sub-Saharan Africa: longitudinal evidence from Burkina Faso and Kenya. BMJ Sex Reprod Health. 2021;47: 252–260. doi: 10.1136/bmjsrh-2020-200944 33579717 PMC7886665

[5] Moreau C, Karp C, Wood S, Williams K, Olaolorun FM, Akilimali P, et al. Trends in fertility intentions and contraceptive practices in the context of COVID-19 in sub-Saharan Africa: insights from four national and regional population-based cohorts. BMJ Open. 2023;13: e062385. doi: 10.1136/bmjopen-2022-062385 36657770 PMC9852736

[6] Wood SN, Karp C, OlaOlorun F, Pierre AZ, Guiella G, Gichangi P, et al. Need for and use of contraception by women before and during COVID-19 in four sub-Saharan African geographies: results from population-based national or regional cohort surveys. The Lancet Global Health. 2021;9: e793–e801. doi: 10.1016/S2214-109X(21)00105-4 34019835 PMC8149322

[7] Assefa N, Sié A, Wang D, Korte ML, Hemler EC, Abdullahi YY, et al. Reported Barriers to Healthcare Access and Service Disruptions Caused by COVID-19 in Burkina Faso, Ethiopia, and Nigeria: A Telephone Survey. Am J Trop Med Hyg. 2021;105: 323–330. doi: 10.4269/ajtmh.20-1619 34161296 PMC8437171

[8] Bolarinwa OA, Ahinkorah BO, Seidu A-A, Ameyaw EK, Saeed BQ, Hagan JE, et al. Mapping Evidence of Impacts of COVID-19 Outbreak on Sexual and Reproductive Health: A Scoping Review. Healthcare (Basel). 2021;9: 436. doi: 10.3390/healthcare9040436 33917784 PMC8068100

[9] Kabagenyi A, Kyaddondo B, Nyachwo EB, Wasswa R, Bwanika JM, Kabajungu E, et al. Disruption in Essential Health Service Delivery: A Qualitative Study on Access to Family Planning Information and Service Utilization During the First Wave of COVID-19 Pandemic in Uganda. Open Access J Contracept. 2022;13: 75–82. doi: 10.2147/OAJC.S360408 35642206 PMC9148575

[10] MOMENTUM. Disrupted Health Services during COVID-19: How Do We Address It? In: USAID MOMENTUM [Internet]. 4 Jan 2021 [cited 6 Jul 2022]. Available: https://usaidmomentum.org/disrupted-health-services-during-covid-19-how-do-we-address-it/

[11] Plotkin MK, Williams KM, Mbinda A, Oficiano VN, Nyauchi B, Walugembe P, et al. Keeping essential reproductive, maternal and child health services available during COVID-19 in Kenya, Mozambique, Uganda and Zimbabwe: analysis of early-pandemic policy guidelines. BMC Public Health. 2022;22: 577. doi: 10.1186/s12889-022-12851-4 35321675 PMC8942058

[12] Shuka Z, Mebratie A, Alemu G, Rieger M, Bedi AS. Use of healthcare services during the COVID-19 pandemic in urban Ethiopia: evidence from retrospective health facility survey data. BMJ Open. 2022;12: e056745. doi: 10.1136/bmjopen-2021-056745 35197352 PMC8882656

[13] Tolu LB, Hurisa T, Abas F, Daba M, Abebe B, Nigatu B, et al. Effect of COVID-19 Pandemic on Safe Abortion and Contraceptive Services and Mitigation Measures: A Case Study From A Tertiary Facility in Ethiopia. Ethiopian Journal of Reproductive Health. 2020;12: 6–6.

[14] Reproductive Health Supplies Coalition. Building Resilient Sexual and Reproductive Health Supply Chains During COVID-19. 2021; 74.

[15] Shapira G, Ahmed T, Drouard SHP, Amor Fernandez P, Kandpal E, Nzelu C, et al. Disruptions in maternal and child health service utilization during COVID-19: analysis from eight sub-Saharan African countries. Health Policy Plan. 2021;36: 1140–1151. doi: 10.1093/heapol/czab064 34146394 PMC8344431

[16] Ameh C, Banke-Thomas A, Balogun M, Makwe CC, Afolabi BB. Reproductive Maternal and Newborn Health Providers’ Assessment of Facility Preparedness and Its Determinants during the COVID-19 Pandemic in Lagos, Nigeria. Am J Trop Med Hyg. 2021;104: 1495–1506. doi: 10.4269/ajtmh.20-1324 33635826 PMC8045608

[17] Banke-Thomas A, Yaya S. Looking ahead in the COVID-19 pandemic: emerging lessons learned for sexual and reproductive health services in low- and middle-income countries. Reproductive Health. 2021;18: 248. doi: 10.1186/s12978-021-01307-4 34906177 PMC8670615

[18] Leight J, Hensly C, Chissano M, Ali L. Short-term effects of the COVID-19 state of emergency on contraceptive access and utilization in Mozambique. PLOS ONE. 2021;16: e0249195. doi: 10.1371/journal.pone.0249195 33765080 PMC7993869

[19] Polis CB, Biddlecom A, Singh S, Ushie BA, Rosman L, Saad A. Impacts of COVID-19 on contraceptive and abortion services in low- and middle-income countries: a scoping review. Sex Reprod Health Matters. 2022;30: 2098557. doi: 10.1080/26410397.2022.2098557 35920612 PMC9351554

[20] Garrib A, Stoops N, McKenzie A, Dlamini L, Govender T, Rohde J, et al. An evaluation of the District Health Information System in rural South Africa. S Afr Med J. 2008;98: 549–552. 18785397

[21] Hagel C, Paton C, Mbevi G, English M. Data for tracking SDGs: challenges in capturing neonatal data from hospitals in Kenya. BMJ Global Health. 2020;5: e002108. doi: 10.1136/bmjgh-2019-002108 32337080 PMC7170465

[22] Maïga A, Jiwani SS, Mutua MK, Porth TA, Taylor CM, Asiki G, et al. Generating statistics from health facility data: the state of routine health information systems in Eastern and Southern Africa. BMJ Global Health. 2019;4: e001849. doi: 10.1136/bmjgh-2019-001849 31637032 PMC6768347

[23] Mremi IR, George J, Rumisha SF, Sindato C, Kimera SI, Mboera LEG. Twenty years of integrated disease surveillance and response in Sub-Saharan Africa: challenges and opportunities for effective management of infectious disease epidemics. One Health Outlook. 2021;3: 22. doi: 10.1186/s42522-021-00052-9 34749835 PMC8575546

[24] Wickremasinghe D, Hashmi IE, Schellenberg J, Avan BI. District decision-making for health in low-income settings: a systematic literature review. Health Policy and Planning. 2016;31: ii12–ii24. doi: 10.1093/heapol/czv124 27591202 PMC5009221

[25] Bradley SEK, Shiras T. Sources for Family Planning in 36 Countries: Where Women Go and Why it Matters. Rockville, M.D.: Sustaining Health Outcomes through the Private Sector Plus Project, Abt Associates; 2020. Available: https://shopsplusproject.org/sites/default/files/resources/Sources%20for%20Family%20Planning%20in%2036%20Countries-Where%20Women%20Go%20and%20Why%20it%20Matters.pdf

[26] Mangone E, Romorini S. Private Sector Engagement in National Health Management Information Systems: Barriers, Strategies, and Global Case Studies. Rockville, M.D.: Sustaining Health Outcomes through the Private Sector Plus Project, Abt Associates; 2021. Available: https://shopsplusproject.org/sites/default/files/resources/Private%20Sector%20Engagement%20in%20National%20HMIS_Barriers,%20Strategies,%20and%20Global%20Case%20Studies.pdf

[27] Mathieu E, Ritchie H, Rodés-Guirao L, Appel C, Giattino C, Hasell J, et al. Coronavirus Pandemic (COVID-19). Our World in Data. 2020 [cited 9 Jan 2023]. Available: https://ourworldindata.org/coronavirus

[28] Reproductive Health Supplies Coalition. Harmonized Suite of Indicators to Measure Stockouts and Availability of Contraceptives version 1.0. Arlington, Va.: JSI Research and Training Institute; 2015 Aug. Available: https://www.rhsupplies.org/uploads/tx_rhscpublications/Harmonized_Suite_of_Indicators.pdf

[29] Barden-O’Fallon J, Ijdi R-E. Need for Standardized Measure of Modern Method Availability: Assessment of Indicators Using Health Facility Data from Three Country Contexts. Studies in Family Planning. 2023;54: 251–263. doi: 10.1111/sifp.12220 36692830

[30] Muhoza P, Koffi AK, Anglewicz P, Gichangi P, Guiella G, OlaOlorun F, et al. Modern contraceptive availability and stockouts: a multi-country analysis of trends in supply and consumption. Health Policy and Planning. 2021;36: 273–287. doi: 10.1093/heapol/czaa197 33454786 PMC8058948

[31] Peters MA, Ahmed T, Azais V, Amor Fernandez P, Baral P, Drouard S, et al. Resilience of front-line facilities during COVID-19: evidence from cross-sectional rapid surveys in eight low- and middle-income countries. Health Policy and Planning. 2023; czad032. doi: 10.1093/heapol/czad032 37256762 PMC11318646

[32] Balogun M, Banke-Thomas A, Sekoni A, Boateng GO, Yesufu V, Wright O, et al. Challenges in access and satisfaction with reproductive, maternal, newborn and child health services in Nigeria during the COVID-19 pandemic: A cross-sectional survey. PLoS One. 2021;16: e0251382. doi: 10.1371/journal.pone.0251382 33961682 PMC8104439

[33] Adelekan B, Goldson E, Abubakar Z, Mueller U, Alayande A, Ojogun T, et al. Effect of COVID-19 pandemic on provision of sexual and reproductive health services in primary health facilities in Nigeria: a cross-sectional study. Reproductive Health. 2021;18: 166. doi: 10.1186/s12978-021-01217-5 34348757 PMC8334336

[34] Brunie A, Austin G, Arkin J, Archie S, Amongin D, Ndejjo R, et al. Women’s Experiences With Family Planning Under COVID-19: A Cross-Sectional, Interactive Voice Response Survey in Malawi, Nepal, Niger, and Uganda. Glob Health Sci Pract. 2022;10: e2200063. doi: 10.9745/GHSP-D-22-00063 36041839 PMC9426982

[35] Chekol BM, Muluye S, Sheehy G. Impacts of COVID-19 on reproductive health service provision, access, and utilization in Ethiopia: Results from a qualitative study with service users, providers, and stakeholders. PLOS Glob Public Health. 2023;3: e0001735. doi: 10.1371/journal.pgph.0001735 36963081 PMC10035746

[36] Shikuku DN, Nyaoke IK, Nyaga LN, Ameh CA. Early indirect impact of COVID-19 pandemic on utilisation and outcomes of reproductive, maternal, newborn, child and adolescent health services in Kenya: A cross-sectional study. African Journal of Reproductive Health. 2021;25. Available: https://www.ajrh.info/index.php/ajrh/article/view/3035 doi: 10.29063/ajrh2021/v25i6.9 37585823

[37] Siedner MJ, Kraemer JD, Meyer MJ, Harling G, Mngomezulu T, Gabela P, et al. Access to primary healthcare during lockdown measures for COVID-19 in rural South Africa: a longitudinal cohort study. medRxiv. 2020; 2020.05.15.20103226. doi: 10.1101/2020.05.15.20103226 33020109 PMC7536636

[38] Makumbi F, Kibira SPS, Giibwa L, Polis C, Giorgio M, Segawa P, et al. Access to Contraceptive Services Among Adolescents in Uganda During the COVID-19 Pandemic. 2021 [cited 12 May 2023]. Available: https://www.guttmacher.org/report/impact-covid-19-on-adolescent-srh-uganda

[39] Kassie A, Wale A, Yismaw W. Impact of Coronavirus Diseases-2019 (COVID-19) on Utilization and Outcome of Reproductive, Maternal, and Newborn Health Services at Governmental Health Facilities in South West Ethiopia, 2020: Comparative Cross-Sectional Study. Int J Womens Health. 2021;13: 479–488. doi: 10.2147/IJWH.S309096 34040456 PMC8141395

