# Supplementary material for: Family planning service disruptions in the first two years of the COVID-19 pandemic: Evidence from health facilities in seven low- and middle-income countries
Source: PLOS Glob Public Health. 2024 Jan 5;4(1):e0002435. doi: 10.1371/journal.pgph.0002435 (PMC10769091; doi:10.1371/journal.pgph.0002435)
Supplement: S1 Checklist — (DOCX) [file pgph.0002435.s001.docx]

Inclusivity in global research

PLOS’ policy on inclusivity in global research aims to improve transparency in the reporting of research performed outside of researchers’ own country or community and ensures that PLOS publications reporting global research adhere to high standards for research ethics and authorship. Authors of relevant research articles may be asked to complete the questionnaire below, which outlines ethical, cultural, and scientific considerations specific to inclusivity in global research. This questionnaire may be requested when researchers have travelled to a different country to conduct research, if research uses samples collected in another country, research with Indigenous populations or their lands, or if research is on cultural artefacts. Researchers travelling to another country solely to use laboratory equipment will not normally be required to complete the questionnaire. However, the questionnaire can be requested at the journal’s discretion for any submission – if you have been requested to complete this questionnaire by the PLOS journal you submitted to, please do so.

Please complete the questionnaire below and include this as a Supporting Information file with your manuscript. Note that if your paper is accepted for publication, this checklist will be published with your article in the supporting information files. Please ensure that you reference the checklist in the main body of your manuscript. We suggest adding a subsection ‘Inclusivity in global research’ to your Methods section and adding the following sentence: “Additional information regarding the ethical, cultural, and scientific considerations specific to inclusivity in global research is included in the Supporting Information (SX Checklist)”

The questions have been designed to be applicable to a wide range of study types, and there are subsections for both human subjects research and non-human subjects research. If any of the questions are not relevant to your research please mark them as “N/A” as appropriate.

**Ethical considerations, permits and authorship**

*This section is applicable to all research types.*

Provide details as to who granted permissions and/or consent for the study to take place in the Methods section of your manuscript. This should include the names of **all** ethics boards, governmental organizations, community leaders or other bodies that provided approval for the study. If individuals provided approval refer to these people by their role or title but do not list their name(s).

*Reported on page number: 6*

*“Trained data collectors in each geography conducted the facility surveys. Per health facility and at each time point (if applicable), the facility manager, main administrator, or a family planning service provider provided verbal informed consent and responded to a series of questions about FP service delivery and operational challenges experienced during COVID-19. Data on contraceptive method provision within each facility were collected directly from the facility’s family planning register, recorded by the facility’s data supervisor. Surveys lasted approximately 30-75 minutes. Ethical approval for the data collection activities was provided by in-country review boards, including the Ethics Committee for Health Research at the Ministry of Health and Ministry of Higher Education, Scientific Research and Innovation (Burkina Faso; N/Refs: A018-2019; A14-2020/CEIRES; ISSP/DA/GG/062/2021); Comité National d’Ethique des Sciences de la Vie et de la Santté at the Ministry of Health of Côte d’Ivoire (N/Refs: 053-2-/MSHP/CNESVS-km; 250-21/MSHP/NCESVS-km; 128-22/MSHPCMU/CNESVS-kp); University of Kinshasa School of Public Health (DRC) (N/Refs: ESP/CE/030B/2019; ESP/CE/78/2020; ESP/CE/160/2020; ESP/CE/159B/2021); Kenyatta National Hospital-University of Nairobi Scientific Ethics Review Committee (N/Refs: KHN-ERC/A/412; KHN-ERC/A/150; KHN-ERC/Mod&SAE/172); Lagos State University Teaching Hospital Research Ethics Committee (N/Refs: LREC/06/10/1276) and Kano State Health Research Ethics Committee of the Ministry of Health and the Research Ethics Committee of the Aminu Kano Teaching Hospital in Kano (Nigeria) (N/Refs: MOH/Off/797/TI/1487; MOH/Off/797/TI/2006; MOH/Off/797/TI/2096; SHREC/2021/2880); Indian Institute of Health Management Research University Institutional Committee for Ethics and Review of Research (Rajasthan) (N/Ref: 0990-0279), and Makerere University School of Public Health (Uganda) (N/Ref: HDREC805). The SDP survey was deemed IRB-exempt as non-human subjects research by the Johns Hopkins Bloomberg School of Public Health.”*

If there were any deviations from the study protocol after approval was obtained please provide details of these changes in the Methods section of your manuscript.

*Reported on page number: N/A*

*No deviations from the study protocol occurred after approval was obtained from the ethical review boards, thus, this information is not reported in the Methods section of the manuscript.*

Did this study involve local collaborators that are residents of the country where the research was conducted or members of the community studied? If you do not have any authors from said communities, please provide an explanation for this below.

*Yes, this study involved local collaborators that are residents of the country where the research was conducted. These research team members are included as co-authors of this research and manuscript.*

Everyone listed as an author should meet PLOS’ criteria for authorship and all individuals who meet these criteria should be included in the author byline, rather than the acknowledgements. For further information please see the journal’s Authorship Policy.

*We confirm all authors meet PLOS’ criteria for authorship and all individuals who meet these criteria are included in the author byline by name, rather than in the acknowledgements.*

**Human subjects research (e.g. health research, medical research, cross-cultural psychology)**

Did you obtain written informed consent from a representative of the local community or region before the research took place? How did you establish who speaks for the community? Details of written informed consent obtained from study participants should be reported separately in the Methods section of your manuscript.

*This research was conducted as part of the broader Performance Monitoring for Action (PMA) project, which has implemented population- and facility-based research in the study geographies for nearly a decade. As such, this study leveraged the existing network of research collaborations within each geography before the research took place; this process did not explicitly include informed consent from a representative of the local community or region, given the focus on facility-based data, not individuals’ experiences. While the Service Delivery Point (SDP) survey used for this study was deemed IRB-exempt as non-human subjects research by the Johns Hopkins Bloomberg School of Public Health, verbal informed consent was obtained from facility manager, main administrator, or a family planning service providers who participated in the survey on behalf of the facility at-large. This information in reported in the Methods section of the manuscript on pg. 6.*

How did members of the local community provide input on the aims of the research investigation, its methodology, and its anticipated outcome(s)?

*Members of the local community, specifically representatives from the national or regional geographies where the SDP surveys were administered, provided input on the aims of the research investigation in-line with national or regional interests in understanding COVID-19’s impact on family planning services and utilization. The methodology of the SDP survey was implemented following the standard PMA protocol that has been approved and used in study geographies throughout the duration of the PMA project.*

When engaging with the local community, how did you ensure that the informed consent documents and other materials could be understood by local stakeholders?

*As with all PMA questionnaires and data collection, our study team ensured that the informed consent documents and other materials could be understood by local stakeholders by following a standard survey tool testing and piloting process. This process is used to ensure that all study materials are understood, and adapted as necessary, to be understood and applicable to the culture and context of each study geography.*

Will the findings of the research be made available in an understandable format to stakeholders in the community where the study was conducted (e.g. via a presentation, summary report, copies of publications, etc.)? Please provide details of how this will be achieved.

*Yes, the findings of the research have been made available in an understandable format to stakeholders in the geographies where the study was conducted. Additionally, key findings have been disseminated more broadly through presentations and summary reports, including publicly accessible materials.*

**Non-human subjects research using specimens/ animals collected as part of the study, or those housed in archival collections. Examples include archaeology, paleontology, botany and zoology.**

Did the permission you obtained from a local authority to perform the study include an agreement on access to outputs and benefit sharing? This may include procedures to enable fair distribution of the benefits and resources arising from the research performed. Please include any details of Prior Informed Consent and Benefit Sharing Agreements obtained. These may be required by field-specific regulations, for example the Convention on Biological Diversity (CBD) and the associated Nagoya Protocol.

*N/A*

If the material used in your study was imported, please A) provide the year it was imported and B) indicate whether permits were obtained to import/export the materials used, C) provide details of any permits obtained. If this information is not available, please indicate this.

*N/A*

If you used archival specimens, please state how the material used in your study was acquired by the institute it is held in and provide details of any permits obtained for the original excavations/ sample collection. If this information is not available, please indicate this.

*N/A*

How was the potential cultural significance of the materials collected in your study to local communities considered in your research design? Were Indigenous peoples and/or local researchers and institutions involved with archaeological excavations / collection of specimens? If so, please provide a description of their involvement.

*N/A*

If your manuscript includes photographs of human remains please indicate whether authors obtained permission from descendants or affiliated cultural communities to do so.

*N/A*
